# A Citrus Fruit Extract High in Polyphenols Beneficially Modulates the Gut Microbiota of Healthy Human Volunteers in a Validated In Vitro Model of the Colon

**DOI:** 10.3390/nu13113915

**Published:** 2021-11-01

**Authors:** Mônica Maurer Sost, Sanne Ahles, Jessica Verhoeven, Sanne Verbruggen, Yala Stevens, Koen Venema

**Affiliations:** 1 Centre for Healthy Eating & Food Innovation (HEFI), Campus Venlo, Maastricht University, Villafloraweg 1, 5928 SZ Venlo, The Netherlands; m.maurersost@maastrichtuniversity.nl (M.M.S.); jessica.verhoeven@maastrichtuniversity.nl (J.V.); s.verbruggen@maastrichtuniversity.nl (S.V.); 2 BioActor B.V., 6229 GS Maastricht, The Netherlands; sanne.ahles@bioactor.com (S.A.); yala.stevens@bioactor.com (Y.S.); 3 Department of Nutrition and Movement Sciences, School of Nutrition and Translational Research in Metabolism (NUTRIM), Maastricht University, 6200 MD Maastricht, The Netherlands; 4 Department of Internal Medicine, School of Nutrition and Translational Research in Metabolism (NUTRIM), Maastricht University, 6200 MD Maastricht, The Netherlands

**Keywords:** *Roseburia*, Citrus Fruit Extract, gut microbiota, short-chain fatty acids (SCFA), in vitro model, TIM-2

## Abstract

The effect of a Citrus Fruit Extract high in the polyphenols hesperidin and naringin (CFE) on modulation of the composition and activity of the gut microbiota was tested in a validated, dynamic in vitro model of the colon (TIM-2). CFE was provided at two doses (250 and 350 mg/day) for 3 days. CFE led to a dose-dependent increase in *Roseburia*, *Eubacterium ramulus*, and *Bacteroides eggerthii*. There was a shift in production of short-chain fatty acids, where acetate production increased on CFE, while butyrate decreased. In overweight and obesity, acetate has been shown to increase fat oxidation when produced in the distal gut, and stimulate secretion of appetite-suppressive neuropeptides. Thus, the data in the in vitro model point towards mechanisms underlying the effects of the polyphenols in CFE with respect to modulation of the gut microbiota, both in composition and activity. These results should be confirmed in a clinical trial.

## 1. Introduction

The gastrointestinal tract (GI tract) consists of various organs, such as the stomach, small intestine, and large intestine. Within the GI tract, nutrients pass various chemical (stomach acidity, intestinal bile) and physical (mucus layer, intestinal epithelial cells) barriers and are digested by salivary, gastric, and pancreatic enzymes in order to be absorbed into the blood [1]. These barriers play a crucial role in maintaining intestinal homeostasis by providing protection against pathogens and simultaneously preserving a symbiotic relationship with commensal microorganisms. The collection of commensal microorganisms in the gut is called the gut microbiota and is unique for each individual. It consists of bacteria, fungi and yeasts, viruses and bacteriophages, and protozoa [2]. The four main bacterial components comprise the phyla Firmicutes, Bacteroidetes, Actinobacteria, and Proteobacteria. Differences in gut microbiota composition are observed due to genetics, age, diet, geographic origin, medication use, and disease [3,4]. Over the last few decades, the role of the gut microbiota in numerous diseases and disorders has been shown, not only including disease in the gut, such as inflammatory bowel disease and colon cancer, but also elsewhere in the body, such as allergy of skin and lungs, obesity, and brain-related disorders [5,6]. Overall, high microbiota diversity, stability of gut microbiota composition, and a low Firmicutes-to-Bacteroidetes ratio are thought to be beneficial [7], although for the latter not all data point into the same direction [8] and may depend on the type of experiment in which the data were obtained: in vitro experiment, animal, or clinical trial. 

The dynamic TNO in vitro model of the colon (TIM-2) system [9] has been extensively used to assess the diversity and composition of the microbiota (for a review see [10]). It is a validated and computer-controlled model of the human proximal colon that contains various components such as a dialysis system, temperature control, and peristaltic movements to regulate and simulate the conditions in the large intestine and has been described in detail before. It consists of four units that can be run in parallel (for a schematic of one unit see Figure S1). In particular, the dialysis system, which prevents the accumulation of microbial metabolites that would otherwise inhibit the microbiota, allows the model to closely simulate the human proximal colon [9,10].

Apart from microbiota composition, the activity of the microbiota is considered important. The metabolites that are produced upon fermentation of undigestible/undigested food components are absorbed by the colonocytes and interact with the host. Dietary fibers are fermented by gut bacteria into short-chain fatty acids (SCFA). The most abundant are acetate, propionate, and butyrate, accounting for approximately 90–95% of all SCFA [11]. These three SCFA are present in a molar ratio of approximately 60:20:20, respectively, and are beneficial for health through immune modulation, anti-inflammatory properties, and improved carbohydrate and lipid metabolism [12]. All SCFA have been shown to be important energy sources for the body, depending on location within the body. For example, butyrate is an energy source for colonocytes and is extremely important in colonic barrier function, while acetate is an energy substrate for liver, muscle, and brain [13,14,15,16,17].

Besides our own genetic make-up, diet is one of the drivers that explains most of the variation between individuals in terms of microbiota composition. A lot of research in the past few decades has focused on modulation of the composition or activity of the gut microbiota by use of functional foods such as flavonoids. These are a class of natural substances, mainly found in fruits and vegetables, that exert beneficial effects on health [18]. Anti-inflammatory, anti-oxidative, anti-cancer, and anti-viral properties have been described for various flavonoids such as hesperidin and naringin [19,20,21]. Both compounds are found mainly in citrus fruits and belong to the subclass flavanones. Hesperidin and naringin are both metabolized by the intestinal microbiota to their aglycones hesperetin and naringenin, respectively. These aglycones have been shown to have beneficial effects on barrier function and inflammation in the intestine [22]. Various in vitro and animal studies have shown positive effects of hesperetin and naringenin on SCFA production and microbiota composition [23,24,25]. Previously, we performed a clinical study on 50 individuals with daily supplementation of 500 mg of a Citrus Fruit Extract (CFE) or placebo for 12 weeks, observing a relative increase in butyrate levels (unpublished data). However, the effect of CFE on the gut microbiota composition has not yet been investigated. Therefore, the aim of this study was to assess the effect of daily supplementation with CFE on gut microbiota composition and activity using the validated, computer-controlled Netherlands Organisation for Applied Scientific Research (TNO) in vitro model of the proximal colon (TIM-2).

## 2. Materials and Methods

### 2.1. Study Product

CFE containing 88.2% hesperidin and 6.5% naringin (MicrobiomeX^®^, BioActor BV, Maastricht, The Netherlands) was added in two different concentrations (250 mg extract/day; 350 mg extract/day) to the standard ileal efflux medium (SIEM) for the TIM-2 experiment. SIEM was included as a control condition and consisted of starch, pectin, xylan, arabinogalactan, amylopectin, protein, vitamins, salts, Tween 80, and ox bile, as described by Cuevas Tena et al. [26].

### 2.2. Collection and Preparation of Fecal Samples

Fecal samples from healthy volunteers (n = 7; 3 M, 4 F, average age = 32 years) were collected and homogenized under anaerobic conditions to create a standardized microbiota pool according to Aguirre et al. [27]. The fecal slurry was snap-frozen in liquid nitrogen and stored at −80 °C until further use. Before inoculation, 4 tubes of 30 mL of fecal slurry each were thawed for 1 h at 37 °C and subsequently mixed with prereduced dialysate to a total volume of 250 mL.

### 2.3. Experimental Set Up and the TIM-2 In Vitro Model

Each TIM-2 unit was inoculated with 60 mL of the standardized microbiota pool, as described in Section 2.2, and 60 mL prereduced dialysate. Subsequently, SIEM was administered to each unit (2.5 mL/h) for an adaptation period of 16 h. Thereafter, the units were continuously supplemented for 72 h with a constant flow of SIEM (2.5 mL/h) in three conditions: SIEM (control), SIEM + 250 mg citrus extract/day, SIEM + 350 mg citrus extract/day. Throughout the experiment, lumen samples were removed from the units after 24 and 48 h to simulate passage from the proximal to the distal colon. Lumen and dialysate samples were obtained at 0, 24, 48, and 72 h, and analyzed for metabolite production (see Section 2.4) and microbiota composition and activity.

### 2.4. SCFA, BCFA, and Organic Acid Production

SCFA, branched-chain fatty acids (BCFA), and organic acid production was analyzed through ion exclusion chromatography by Brightlabs (Venlo, The Netherlands) as described previously [26]. Briefly, lumen and dialysate samples were centrifuged at 14,000 rpm for 10 min, filtered, and diluted with 1.5 mM sulfuric acid. Next, 10 μL of this solution was added to a column, and analysis was started on an 883 chromatograph (IC, Methorm, Herisa, Switzerland).

### 2.5. Gut Microbiota Composition

Sequencing of the V3-V4 region of the 16S rRNA gene was performed to determine microbiota composition. In short, from the lumen samples, DNA was isolated, amplified with barcoding, pooled, and subsequently sequenced with the Illumina MiSeq sequencing system according to the manufacturer’s instructions (Illumina, Eindhoven, The Netherlands). The Binary Base Call text-based format for storing biological sequence and corresponding quality scores pipeline (BCL2FASTQ, v. 1.8.3, Illumina, San Diego, CA, United States of America) was used to convert the sequences into text-based format for storing biological sequence and corresponding quality score (FASTQ) files after quality checking. Subsequently, Quantitative Insights Into Microbial Ecology 2 (QIIME-2) software was used to analyze the results [28]. Classification of the sequences into amplicon sequence variants (ASVs) was performed using the Silva database (version 132 (available online: https://www.arb-silva.de/documentation/release-132/ acessed on 27 October 2021)) as a reference 16S rRNA database.

### 2.6. Statistical Analysis

The software package R (version 3.6.2, R Foundation for Statistical Computing, Vienna, Austria (R Core Team, 2013; https://cran.r-project.org/bin/windows/base/old/3.6.2/ accessed on 27 October 2021)) was used for statistical analyses. To determine changes in the microbial community composition, various indexes were calculated and the abundances of microbial species in the total microbial community were calculated and shown as relative abundance (RA). Kruskal–Wallis analysis was performed to determine differences in species abundance between the treatment conditions and its dose dependence. The correlation of RA of ASVs and microbial metabolites were calculated with Spearman’s correlation. Multiple comparisons were adjusted with the Benjamini–Hochberg false discovery rate, and q-values (FDR-corrected *p*-values) were considered significant at *q* < 0.20.

## 3. Results

### 3.1. Changes in Microbiota Composition

**Figure 1:**
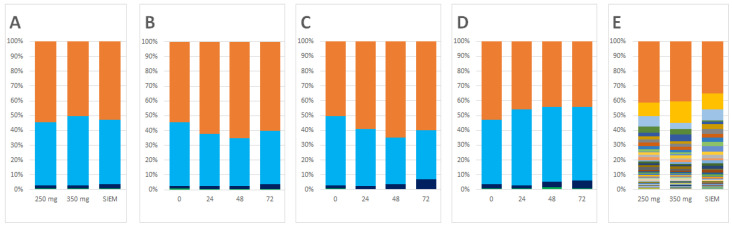
Composition in relative abundance of the samples from the in vitro model. (**A**) Composition at the start of the intervention at the phylum level (standard ileal efflux medium (SIEM)); (**B**) compositional changes over time for the 250 mg Citrus Fruit Extract (CFE) dose; (**C**) compositional changes over time for the 350 mg CFE dose; (**D**) compositional changes over time for the SIEM; (**E**) composition at the start of the intervention at the genus level. At the phylum level, the major phyla are shown: Bacteroidetes (orange); Firmicutes (light blue); Proteobacteria (dark blue). All other taxa were grouped under “Other” (green). For the genus level composition, the legend is provided in Table S1 for the top 25 genera, together with the quantitative data for all genera.

Specific alterations in microbiota compositions between control and CFE addition were investigated. Since the inoculum was standardized by pooling [27], the different experimental conditions commenced with the same microbiota composition after the adaptation period at both the phylum and genus level (Figure 1A,E, respectively). Overall, there was a slight increase in Bacteroidetes and a corresponding decrease in Firmicutes over time for the two CFE doses compared to SIEM (Figure 1B–D).

**Figure 2:**
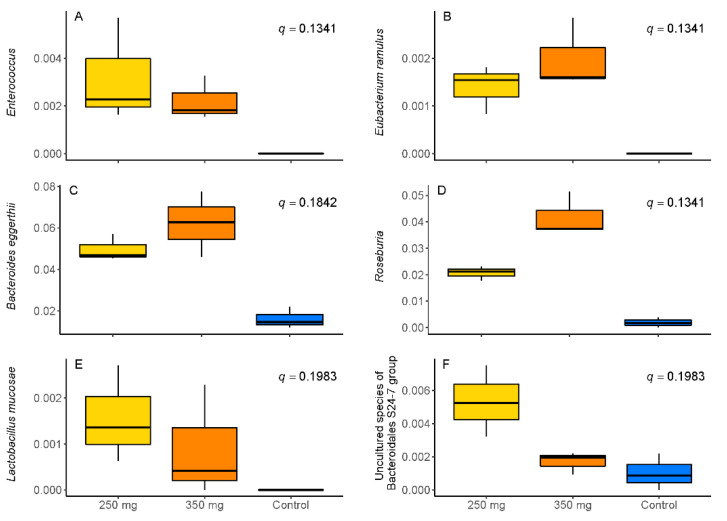
Relative abundance of (**A**) *Enterococcus*; (**B**) *Eubacterium ramulus*; (**C**) *Bacteroides eggerthii*; (**D**) *Roseburia*; (**E**) *Lactobacillus mucosae,* and (**F**) an uncharacterized species of the *Bacteroidales S24-7 group* after supplementation with standard ileal efflux medium (SIEM (control; blue)) or 250 mg (yellow) or 350 mg (orange) of Citrus Fruit Extract.

At the genus level, when compared to control, supplementation with the CFE doses resulted in a significant increase in the relative abundance of the genera *Enterococcus* (*q* = 0.134; Figure 2A) and *Roseburia* (*q* = 0.134; Figure 2D) which both belong to the phylum Firmicutes, despite an overall slight reduction in Firmicutes over time (Figure 1).

In addition, the relative abundance of the species *Eubacterium* (*E.*) *ramulus* (*q* = 0.134; Figure 2B), *Limosilactobacillus* (formerly *Lactobacillus*) (*L.*) *mucosae* (*q* = 0.198; Figure 2E), from the phylum *Firmicutes*, and *Bacteroides* (*B.*) *eggerthii* (*q* = 0.184; Figure 2C) and an uncharacterized species of the Bacteroides S24-7 group (*q* = 0.198; Figure 2F), from the phylum Bacteroidetes, significantly increased after supplementation with CFE.

Roseburia (*q* = 0.134; *p* = 0.04953) showed a dose-dependent increase upon addition of CFE, while *B. eggerthii* (*q* = 0.184, *p* = 0.2752) and *E. ramulus* (*q* = 0.134, *p* = 0.2752) presented a similar trend (Figure 2). In contrast, the uncharacterized species of the *Bacteroides S24-7 group* showed an inverse dose dependency with higher production at the lower dose of CFE, and *L. mucosae* and *Enterococcus* seemed to follow this trend. Apart from these increases in taxa on both doses, a decrease in Ruminococcaceae UCG-014 was observed after CFE feeding, and an increase only with the low dose CFE was observed for Lachnospiraceae UCG-004 and *Bifidobacterium longum* subsp. *longum* (Figure S2).

**Figure 3:**
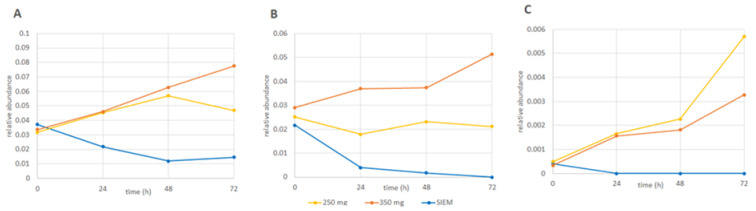
Changes in the relative abundance overtime of (**A**) *B. eggerthii*; (**B**) *Roseburia*, and (**C**) *Enterococcus* for the interventions with 250 mg (yellow) and 350 mg (orange) Citrus Fruit Extract and the control (standard ileal efflux medium (SIEM; blue)). Similar graphs for the other 3 taxa can be found in Figure S3.

Figure 3 shows the changes in relative abundance over time for *B. eggerthii*, *Roseburia*, and *Enterococcus*. Whereas for the two doses of CFE, the RA of these taxa increases over time (or remains relatively the same for the 250 mg dose for Roseburia), the RA of these taxa is reduced in the control (for *Enterococcus* to below the level of detection). This change over time for the uncharacterized species of the *Bacteroidales S24-7 group*, *E. ramulus*, and *L. mucosae* is shown in Figure S3. Essentially similar phenomena as observed for the taxa in Figure 3 are observed for the latter three taxa, although the graph is more inconsistent, with some peaks either up or down (e.g., Figure S3B,C, respectively).

### 3.2. Production of SCFA, BCFA, and Other Organic Acids

**Figure 4:**
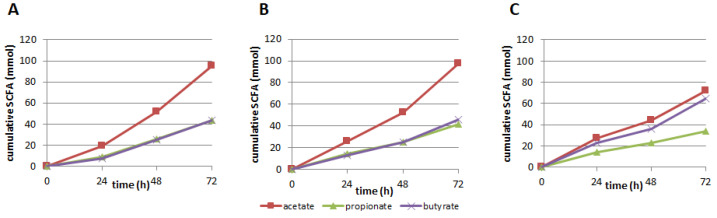
Cumulative production (mmol) of the short-chain fatty acids (SCFA) acetate, propionate, and butyrate at 24 h, 48 h, and 72 h after supplementation with (**A**) 250 mg; (**B**) 350 mg Citrus Fruit Extract, or (**C**) control (standard ileal efflux medium (SIEM)).

After 72 h of continuous supplementation with CFE, cumulative production of the most abundant SCFA acetate, propionate, and butyrate was higher compared to the SIEM control condition for both the 250 and 350 mg CFE addition (Table 1; Figure 4). However, when corrected for the number of carbon-atoms present in acetate (2 carbon atoms), propionate (3 C-atoms), and butyrate (4 C-atoms), it is clear that the CFE primarily led to a shift in production of these acids towards more acetate. The fact that the same number of C-atoms is present in these metabolites (which were the major metabolites produced) is logical, as the amount of carbohydrate provided in each of the experiments was equal. Acetate has the lowest acid logarithmic dissociation constant (pKa) of the three major SCFA and a higher amount of acetate is expected to lead to greater inhibition of pathogenic microorganisms. 

**Figure 5:**
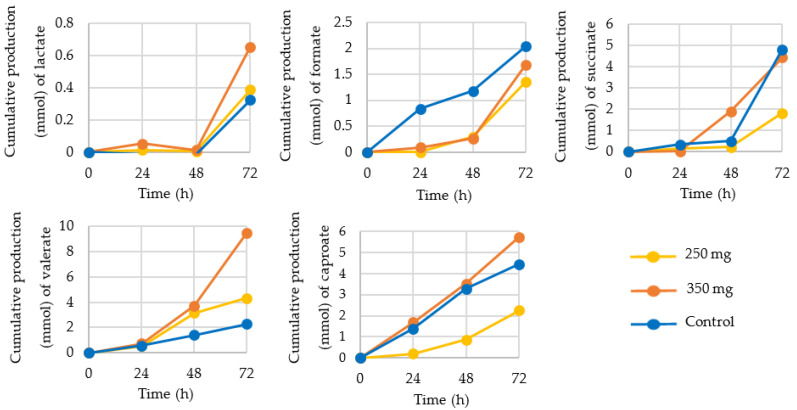
Cumulative production (mmol) of organic acids lactate, formate, succinate, valerate, and caproate at 24, 48, and 72 h after supplementation with 250 mg (yellow) or 350 mg (orange) Citrus Frui Extract or control (standard ileal efflux medium (SIEM; blue)).

**Figure 6:**
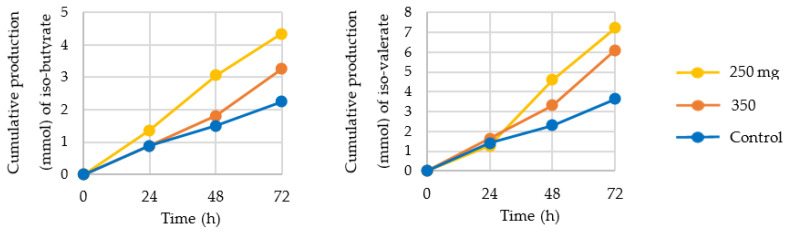
Cumulative production (mmol) of the branched-chain fatty acids (BCFA) iso-butyrate and iso-valerate at 24 h, 48 h, and 72 h after supplementation with 250 mg (yellow) or 350 mg (orange) Citrus Fruit Extract or control (standard ileal efflux medium (SIEM; blue)).

Next to the SCFA acetate, propionate, and butyrate, production of several other organic acids was determined, such as lactate, formate, succinate, valerate, and caproate (Figure 5) as well as the branched-chain fatty acids (BCFA) iso-valerate and iso-butyrate (Figure 6).

Production of lactate, a precursor for propionate, was increased by nearly 100% after 72 h of 350 mg CFE supplementation, compared to control. A similar pattern was observed for valerate production, with a >300% increase after addition of 350 mg/day CFE and an 85% increase after addition of 250 mg/day CFE. Iso-valerate and iso-butyrate production was increased by both concentrations of CFE, while formate, a substrate for acetate production via the Wood-Ljundahl pathway, was decreased. Succinate and caproate production were decreased after 250 mg/day CFE supplementation.

**Figure 7:**
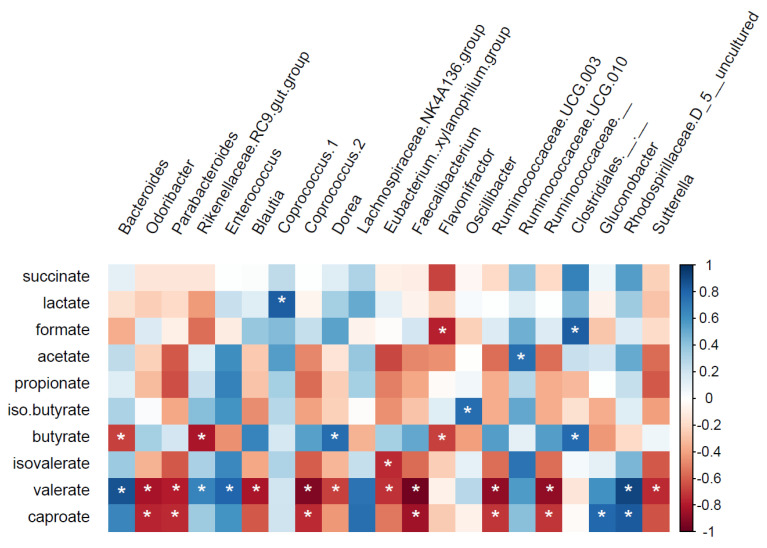
Correlation between metabolite production and specific operational taxonomic unit (OTUs) at genus level for rho values ≥0.75 or ≤−0.75. White asterisks (*) *q* ≤ 0.2; blue: positive correlation; red: negative correlation.

Spearman correlations were performed between metabolite concentrations and specific operational taxonomic units (OTUs) at genus and species levels (Figure 7 and Figure S4). Five butyrate-producing genera (*Dorea*; Figure 7, *Anaerostipes*, *Coprococcus 3*, *Lachnospiraceae ND3007 group*, and *Eubacterium hallii group*; Figure S4) had a significant positive correlation with butyrate production. *Ruminococcaceae UCG-010* (*q* = 0.14, rho = 0.75, Figure 7) and Lactobacillus (*q* = 0.16, rho = 0.74; Figure S4) were positively correlated with acetate and propionate, respectively. *Ruminococcaceae UCG-010* also showed a positive correlation with iso-valerate (*q* = 0.16, rho = 0.73; Figure S4). A negative correlation was found between the genera *Rikenellaceae RC9-gut-group*, an unknown genus from Ruminococcaceae family, *Bacteroides*, and *Flavonifractor* with butyrate production (Figure S4). *Flavonifractor* also had a negative correlation with formate (*q* = 0.09, rho = −0.77; Figure 7), while *Bacteroides* showed a positive correlation with valerate (*q* = 0.01, rho = 0.87, Figure 7). All correlations for lactate and succinate were positive (rho > 0.73), namely with the genera *Coprococcus 1* (Figure 7) and *Dialister*, and an unknown genus from the family Clostridiales vadinBB60-group, respectively (Figure S4). A wide range of OTUs showed negative correlation with both caproate and valerate, including *Faecalibacterium*, *Odoribacter*, *Parabacteroides*, *Coprococcus 2*, *Ruminococcaceae UCG-003*, *Ruminococcaceae* (Figure 7), *Eubacterium oxidoreducens group*, and *Alistipes* (Figure S4).

## 4. Discussion

In the last few decades, the modulation of the composition and activity of the intestinal microbiota by polyphenols, such as flavonoids, has been a topic that has received increasing attention from the scientific community. In this study, we aimed at investigating the effect of supplementation with CFE on gut microbiota composition and activity using a validated, dynamic, computer-controlled in vitro model: TIM-2.

### 4.1. Changes in Microbiota Composition

Changes over time in microbiota composition were found between control and our study product. After CFE supplementation, genera belonging to the phylum *Firmicutes* (i.e., *Enterococcus* and *Roseburia*) and Bacteroidetes (i.e., *Bacteroides*) showed an increase in relative abundance compared to the control. *Enterococcus* has been shown before to be stimulated by polyphenols from grapes in a study with broiler chickens [29]. *Roseburia* has been shown to be increased by polyphenols (amongst others from grape seed [30,31] red apple [32], and tea [33,34]), as well as reduced (e.g., by soy isoflavones [35], decaffeinated green and black tea [36], or pomegranate peels [37].) Moreover, previous studies evaluating microbiota composition in overweight subjects have reported beneficial effects of polyphenol intake through red wine consumption [38] and sorghum bran intake [39] on *Roseburia* growth. *Roseburia* is one of the most abundant intestinal butyrate-producing bacteria and has been linked with a reduction in inflammation and anti-obesity effects [40]. In addition, administration of naringenin in a letrozole-induced polycystic ovary syndrome model in rats has shown a positive impact on the relative abundance of the genus *Roseburia* [41]. Moreover, in a randomized controlled human intervention trial, three weeks of supplementation with virgin olive oil enriched with 500 mg phenolic compounds increased abundance of *Roseburia*, though it did not reach statistical significance [42]. *E. ramulus* has the capacity to metabolize numerous polyphenols, through ring-opening by use of the enzyme chalcone isomerase [43], as well as further reductive metabolism by an NADH-dependent reductase [44,45]. Through these activities and the metabolites produced as a consequence, *E. ramulus* is thought to contribute to alleviation of obesity [46]. *L. mucosae* is one of the well-known bacteria capable of converting the soy isoflavone daidzein into equol and/or O-desmethylangolensin metabolite [47]. Similarly, it may be able to degrade other polyphenols, in casu the CFE used here. Equol, as well as hesperidin, has been linked with bone loss prevention and serum and a decrease in hepatic lipids [48,49]. Lastly, *B. eggerthii* is one of the major phenylpropanoid-derived metabolite producers in the gut, and these metabolites are frequently found upon polyphenol metabolism, although *B. eggerthii* has been shown to produce these from other aromatic substrates, such as phenolic amino acids [50].

The amount of hesperidin in the 250 and 350 mg CFE doses translates into approximately 0.45–0.63 L of orange juice [51]. Not only by the consumption of citrus extract but also by consumption of citrus fruit juice seems to have a positive effect on microbiota composition. In a controlled clinical study, ten healthy women were evaluated after continuous consumption of commercial pasteurized orange juice for two months. The authors showed that orange juice affected the growth of intestinal bacteria (mainly for *Lactobacillus* spp and *Bifidobacterium* spp.). These results suggest a prebiotic effect of daily consumption of orange juice, with a positive effect on the intestinal microbiota and metabolic biomarkers [52]. No data on SCFA were presented in this study.

### 4.2. Production of SCFA, BCFA, and Other Organic Acids

Cumulative SCFA production from both CFE-fed microbiota was higher than the control, mainly in relation to acetate production. Acetate has been shown to be the main energy source for the liver and is also used for lipogenesis in adipose tissue, and oxidized by muscle and brain cells [53,54]. In addition, it has been shown that acetate is capable of reducing appetite via a homeostatic mechanism, through changes in the expression profiles of regulatory neuropeptides that favor appetite suppression [55]. Moreover, in overweight/obese men, acetate has been shown to promote fat oxidation and improve metabolic markers [17]. When cross-feeding mechanisms for conversion of acetate into butyrate do not occur, more acetate is produced and less butyrate. This might explain why butyrate production is observed to be lower after supplementation with CFE, despite observed increases in butyrate-producing taxa. Beneficial properties have also been attributed to propionate (for reviews see [13,56]), although high concentrations have also been linked to autism spectrum disorder [57,58]. Propionate is thought to be mainly metabolized in the liver, where it acts as a precursor for gluconeogenesis, thus influencing metabolic homeostasis [59]. Propionate also interacts with host receptors stimulating the release of satiety signals [60], and may therefore reduce body weight. In addition, immune-modulatory, and in particular, anti-inflammatory effects of propionate have been observed [56]. Of the SCFA, butyrate has been studied the most. In multiple studies, it has been shown to be beneficial, as it is the preferred energy substrate of colonocytes and is thought to reverse colon cancer by induction of differentiation of transformed cells (for a review see [16]). In our study, Spearman correlations have shown that many butyrate-producing taxa correlated with butyrate production. The majority of bacteria with potential to produce butyrate belong to the phylum Firmicutes where the acetyl-coenzyme A (CoA) pathway is the most prevalent [61]. Studies have shown strong co-occurrence between mucolytic bacteria (i.e., *Bacteroides* spp. and *Ruminococcus* spp.) and butyrate producers (i.e., *Anaerostipes caccae* and *Eubacterium* spp.) [62,63,64] as a possible indication that these different microbial groups shared metabolic networks [65].

Regarding to the organic acids and BCFA production, CFE supplementation has shown an increase in cumulative production for lactate, valerate, iso-valerate, and iso-butyrate. The organic acids are usually considered precursors or alternative end-products of acetate, propionate, and butyrate. As a result, variations in production could provide information on cross-feeding mechanisms that take place within the gut. The BCFA are markers for protein fermentation, as they are exclusively derived from the fermentation of the branched-chain amino acids [66]. Valerate and caproate are amongst others involved in cross feeding mechanisms, and are produced by extending propionate and butyrate with acetyl-CoA to produce valerate and caproate, respectively [67,68]. Moreover, production of these metabolites has been linked to protein fermentation [69]. In addition to their effects as precursors, beneficial signaling effects of lactate and valerate have been described recently. For instance, lactate has been shown to play a key role in multiple cellular processes, such as energy regulation, immune tolerance, memory formation, wound healing, ischemic tissue injury, and cancer growth and metastasis [70,71]. Valerate has been shown to protect for eczema, and protects against colitis and necrotic enteritis [72,73,74].

## 5. Conclusions

The citrus extract with 88.2% hesperidin and 6.5% naringin modulated the gut microbiota in a validated, dynamic in vitro model of the colon (TIM-2), both with respect to microbiota composition as well as microbiota activity. *Roseburia*, *Eubacterium ramulus* and *Bacteroides eggerthii* were dose-dependently increased. Metabolically, an increase in acetate was observed. Nevertheless, the three increased taxa did not correlate with acetate production, but *Ruminococcaceae UCG-010* did. Several butyrate-producing taxa correlated with butyrate production, which overall was slightly lowered by the CFE treatment. Several beneficial traits have been ascribed to acetate, including anti-microbial activity against pathogens, increase in fat oxidation and increase in secretion of regulatory neuropeptides that favor appetite suppression. The latter two traits are beneficial for overweight and obese individuals. Although the validated in vitro model that was used has been shown on many cases to be predictive for the in vivo situation, it remains to be seen whether the CFE has a similar effect in human volunteers. This is currently under investigation.

## Figures and Tables

**Table 1:** Cumulative production of acetate, propionate, butyrate, total short-chain fatty acids (SCFA), and the amount of carbon present in these metabolites after 72 h of continuous supplementation with 250 or 350 mg Citrus Fruit Extract versus control (standard ileal efflux medium (SIEM)).

	SCFA	Acetate	Propionate	Butyrate	Total SCFA	(Carbon)
Intervention						
250 mg		94.85	43.76	43.54	182.16	495.16
350 mg		97.41	41.74	45.86	185.01	503.48
SIEM		72.26	34.26	64.74	171.26	506.26

## Data Availability

All raw sequence data and metadata are available from the corresponding author upon reasonable request.

## Supplementary Materials

The following are available online at https://www.mdpi.com/article/10.3390/nu13113915/s1, Figure S1: Schematic representation of one TIM-2 unit. (A) peristaltic compartments with a dialysis membrane inside, (B) pH sensor, (C) NaOH secretion, (D) dialysate system (D1 = dialysate in, D2 = dialysate out), (E) level sensor, (F), gaseous N_2_ inlet, (G) gas outlet, (H) sampling port, (I) feeding syringe with test compound, (J) temperature sensor.; Figure S2: Relative abundance of (A) *Ruminococcaceae UCG-014*, (B) *Bifidobacterium longum* subsp. *longum*, (C) *Lachnospiraceae UCG-004*, after supplementation with SIEM (control; blue) or 250 mg (yellow) or 350 mg (orange) of Citrus Fruit Extract; Figure S3: Changes in the relative abundance over time of (A) *Bacteroidales S24-7 group*, (B) *E. ramulus*, and (C) *L. mucosae* for the interventions with 250 mg (yellow) and 350 mg (orange) CFE and the control (blue); Figure S4: Correlation between metabolite production and specific OTUs at genus level for rho values <0.75 or >−0.75. * *q* ≤ 0.2; blue: positive correlation; red: negative correlation; Table S1: Legend to Figure 1E. Values are relative abundance of the taxa. Top 25 taxa are colored according to Figure 1E.
