# Supplementary material for: A Citrus Fruit Extract High in Polyphenols Beneficially Modulates the Gut Microbiota of Healthy Human Volunteers in a Validated In Vitro Model of the Colon
Source: Nutrients. 2021 Nov 1;13(11):3915. doi: 10.3390/nu13113915 (PMC8619629; doi:10.3390/nu13113915)
Supplement: Supplementary file 1 [file nutrients-13-03915-s001.zip › nutrients-1419940-supplementary.pdf]

Supplementary figures and tables

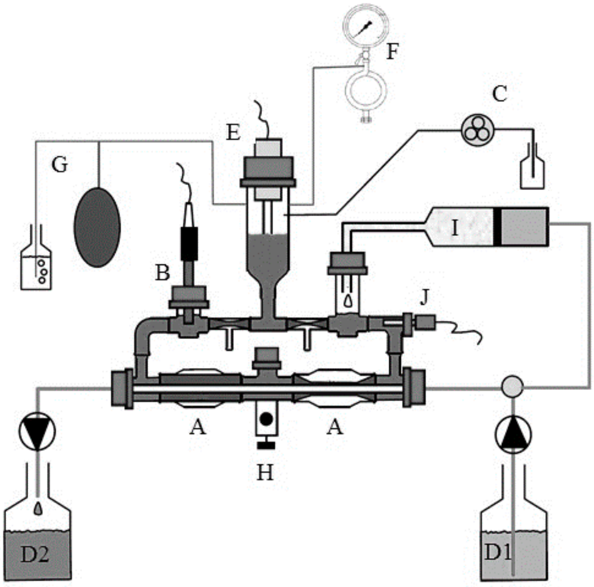

Figure S1. Schematic representation of one TIM-2 unit. (A) peristaltic compartments with a dialysis membrane inside, (B) pH sensor, (C) NaOH secretion, (D) dialysate system (D1=dialysate in, D2=dialysate out), (E) level sensor, (F), gaseous N_2 inlet, (G) gas outlet, H) sampling port, (I) feeding syringe with test compound, (J) temperature sensor.

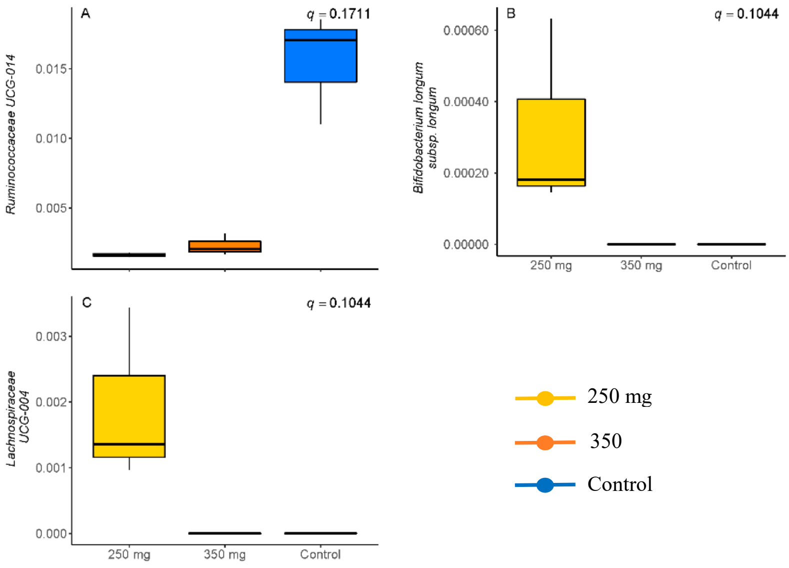

Figure S2. Relative abundance of (A) *Ruminococcaceae UCG-014*, (B) *Bifidobacterium longum* subsp. *longum*, (C) *Lachnospiraceae UCG-004*, after supplementation with Control (SIEM; blue) or 250 mg (yellow) or 350 mg (orange) of citrus fruit extract.

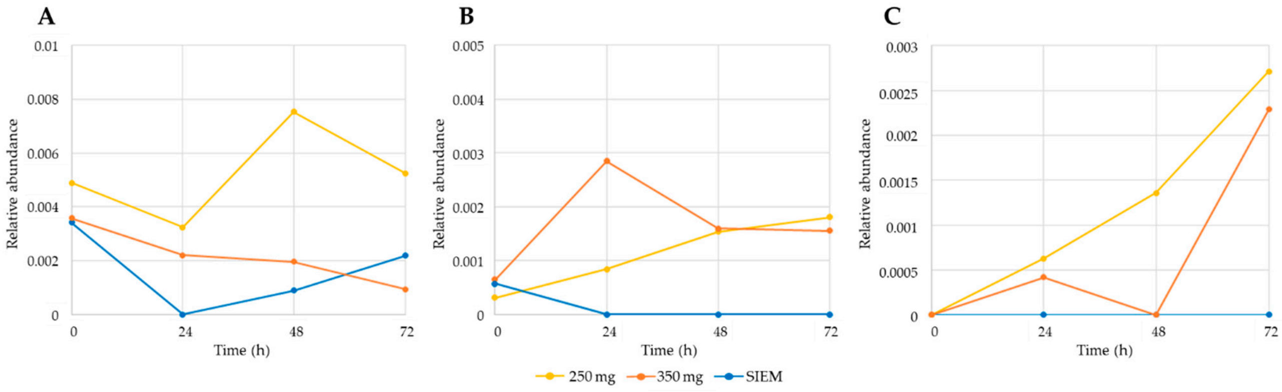

Figure S3. Changes in the relative abundance over time of (A) *Bacteroidales S24-7 group*, (B) *E. ramulus*, and (C) *L. mucosae* for the interventions with 250 mg (yellow) and 350 mg (orange) CFE and the control (blue).

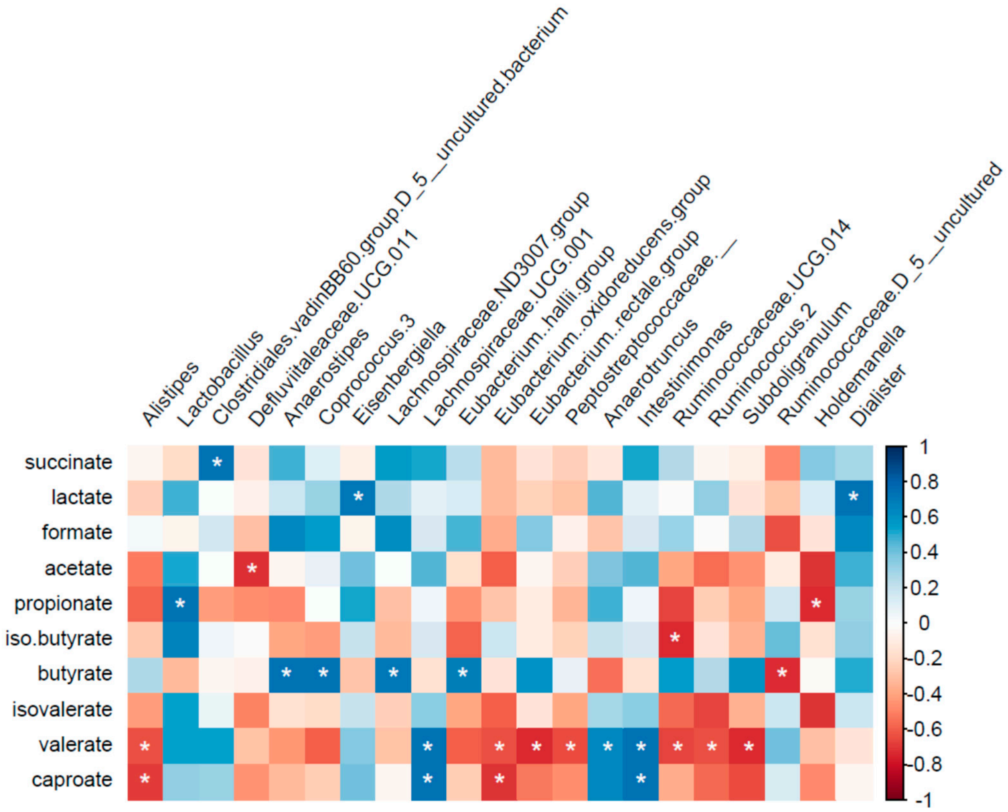

Figure S4. Correlation between metabolite production and specific OTUs at genus level for rho values < 0.75 or > -0.75. **q* ≤ 0.2; blue: positive correlation; red: negative correlation.

Table S1. Legend to Figure 1E. Values are relative abundance of the taxa. Top 25 taxa are colored according to Figure 1E.

	250 mg	350 mg	SIEM
Bacteroidetes; Bacteroidia; Bacteroidales; Bacteroidaceae; Bacteroides	0.411047	0.403427	0.351763
Firmicutes; Clostridia; Clostridiales; Ruminococcaceae; Ruminococcus 2	0.094791	0.147031	0.107315
Bacteroidetes; Bacteroidia; Bacteroidales; Rikenellaceae; Alistipes	0.066985	0.041922	0.074895
Firmicutes; Clostridia; Clostridiales; Lachnospiraceae; Roseburia	0.043281	0.036543	0.008694
Firmicutes; Clostridia; Clostridiales; Lachnospiraceae; __	0.023377	0.042614	0.014196
Firmicutes; Clostridia; Clostridiales; Lachnospiraceae; Blautia	0.023251	0.019607	0.035694
Bacteroidetes; Bacteroidia; Bacteroidales; Prevotellaceae; uncultured	0.019753	0.020737	0.030783
Firmicutes; Clostridia; Clostridiales; Ruminococcaceae; Faecalibacterium	0.025793	0.021594	0.023475
Bacteroidetes; Bacteroidia; Bacteroidales; Prevotellaceae; Prevotella 7	0.017162	0.015441	0.032341
Firmicutes; Clostridia; Clostridiales; Lachnospiraceae; Dorea	0.017715	0.013491	0.027509
Firmicutes; Clostridia; Clostridiales; Lachnospiraceae; [Eubacterium] hallii group	0.005662	0.008395	0.039293
Firmicutes; Clostridia; Clostridiales; Lachnospiraceae; Lachnospiraceae NK4A136 group	0.016759	0.018185	0.017158
Firmicutes; Clostridia; Clostridiales; Ruminococcaceae; Ruminococcaceae UCG-014	0.015778	0.00989	0.02483
Firmicutes; Clostridia; Clostridiales; Ruminococcaceae; Ruminococcaceae UCG-002	0.020332	0.016326	0.012745
Firmicutes; Clostridia; Clostridiales; Ruminococcaceae; __	0.008782	0.010218	0.021992
Firmicutes; Clostridia; Clostridiales; Christensenellaceae; Christensenellaceae R-7 group	0.013563	0.011066	0.016063
Proteobacteria; Betaproteobacteria; Burkholderiales; Alcaligenaceae; Sutterella	0.008883	0.007137	0.020505
Firmicutes; Clostridia; Clostridiales; Lachnospiraceae; [Eubacterium] eligens group	0.016407	0.01463	0.004819
Firmicutes; Negativicutes; Selenomonadales; Acidaminococcaceae; Phascolarctobacterium	0.017212	0.012916	0.005021
Firmicutes; Clostridia; Clostridiales; Ruminococcaceae; Ruminococcaceae UCG-003	0.009864	0.009207	0.013846
Bacteroidetes; Bacteroidia; Bacteroidales; Porphyromonadaceae; Parabacteroides	0.008455	0.007402	0.012453
Bacteroidetes; Bacteroidia; Bacteroidales; Porphyromonadaceae; Barnesiella	0.007297	0.005342	0.014787
Firmicutes; Clostridia; Clostridiales; Ruminococcaceae; Ruminococcaceae UCG-005	0.007927	0.006162	0.003887
Firmicutes; Clostridia; Clostridiales; Lachnospiraceae; Butyrivibrio	0.007474	0.006782	0.00236
Firmicutes; Clostridia; Clostridiales; Lachnospiraceae; Coprococcus 3	0.005586	0.005305	0.002813
Firmicutes; Negativicutes; Selenomonadales; Veillonellaceae; Dialister	0.00463	0.003245	0.005178
Proteobacteria; Betaproteobacteria; Burkholderiales; Alcaligenaceae; Parasutterella	0.004781	0.003464	0.00437
Firmicutes; Clostridia; Clostridiales; Ruminococcaceae; uncultured	0.004152	0.005898	0.002009
Firmicutes; Clostridia; Clostridiales; Ruminococcaceae; Ruminococcaceae NK4A214 group	0.004278	0.00319	0.003971

Firmicutes; Clostridia; Clostridiales; Lachnospiraceae; Lachnospiraceae UCG-004	0.004278	0.004649	0.002394
Bacteroidetes; Bacteroidia; Bacteroidales; Bacteroidales S24-7 group; uncultured bacterium	0.004982	0.003509	0.001752
Firmicutes; Clostridia; Clostridiales; Ruminococcaceae; Subdoligranulum	0.004001	0.004111	0.001916
Proteobacteria; Gammaproteobacteria; Enterobacteriales; Enterobacteriaceae; __	0.002743	0.004567	0.001991
Firmicutes; Clostridia; Clostridiales; Lachnospiraceae; Lachnoclostridium	0.002567	0.002552	0.003198
Firmicutes; Clostridia; Clostridiales; Lachnospiraceae; [Eubacterium] rectale group	0.002516	0.003874	0.00192
Firmicutes; Clostridia; Clostridiales; Lachnospiraceae; Lachnospiraceae ND3007 group	0.00161	0.002124	0.004095
Actinobacteria; Actinobacteria; Bifidobacteriales; Bifidobacteriaceae; Bifidobacterium	0.001585	0.002106	0.003482
Bacteroidetes; Bacteroidia; Bacteroidales; Porphyromonadaceae; Odoribacter	0.001711	0.001057	0.003638
Bacteroidetes; Bacteroidia; Bacteroidales; Rikenellaceae; Rikenellaceae RC9 gut group	0.001258	0.001094	0.00321
Verrucomicrobia; Verrucomicrobiae; Verrucomicrobiales; Verrucomicrobiaceae; Akkermansia	0.002567	0.001777	0.000951
Firmicutes; Clostridia; Clostridiales; Peptostreptococcaceae; __	0.001158	0.001522	0.002468
Firmicutes; Clostridia; Clostridiales; Defluviitaleaceae; Defluviitaleaceae UCG-011	0.002693	0.001395	0.000924
Actinobacteria; Coriobacteriia; Coriobacteriales; Coriobacteriaceae; Collinsella	0.001661	0.002425	0.000752
Bacteroidetes; Bacteroidia; Bacteroidales; __; __	0.001837	0.001194	0.001645
Tenericutes; Mollicutes; Anaeroplasmatales; Anaeroplasmataceae; Anaeroplasma	0.001535	0.001942	0.000862
Firmicutes; Clostridia; Clostridiales; Clostridiaceae 1; Clostridium sensu stricto 1	0.001535	0.00165	0.000762
Firmicutes; Clostridia; Clostridiales; Lachnospiraceae; Lachnospira	0.00161	0.001167	0.000828
Firmicutes; Clostridia; Clostridiales; Lachnospiraceae; [Ruminococcus] torques group	0.00156	0.001276	0.000618
Firmicutes; Clostridia; Clostridiales; Clostridiales vadinBB60 group; uncultured bacterium	0.001007	0.001149	0.001189
Firmicutes; Erysipelotrichia; Erysipelotrichales; Erysipelotrichaceae; Turicibacter	0	0.000902	0.002152
Proteobacteria; Alphaproteobacteria; Rhodospirillales; Rhodospirillaceae; uncultured	0.001082	0.001121	0.000841
Firmicutes; Clostridia; Clostridiales; Lachnospiraceae; uncultured	0.001258	0.000966	0.000697
Firmicutes; Clostridia; Clostridiales; Ruminococcaceae; Ruminiclostridium 9	0.000403	0.000866	0.001609
Firmicutes; Clostridia; Clostridiales; Ruminococcaceae; Ruminiclostridium 6	0.001661	0.000829	0.00033
Firmicutes; Clostridia; Clostridiales; Ruminococcaceae; Ruminococcus 1	0.001309	0.000829	0.00068
Firmicutes; Clostridia; Clostridiales; Lachnospiraceae; [Eubacterium] xylanophilum group	0	0.001258	0.001549
Firmicutes; Clostridia; Clostridiales; Lachnospiraceae; Coprococcus 2	0.001636	0.000829	0.000309
Firmicutes; Clostridia; Clostridiales; Lachnospiraceae; [Ruminococcus] gauvreauii group	0	0.001121	0.00165
Firmicutes; Erysipelotrichia; Erysipelotrichales; Erysipelotrichaceae; Holdemanella	0.001183	0.001094	0.000405
Firmicutes; Clostridia; Clostridiales; Lachnospiraceae; Coprococcus 1	0.001107	0.001057	0.000477

Firmicutes; Clostridia; Clostridiales; Ruminococcaceae; [Eubacterium] coprostanoligenes group	0.001535	0.000729	0.000357
Bacteroidetes; Bacteroidia; Bacteroidales; Porphyromonadaceae; uncultured	0.001183	0.000784	0.000292
Firmicutes; Clostridia; Clostridiales; Ruminococcaceae; Ruminococcaceae UCG-013	0.000403	0.000848	0.000976
Firmicutes; Clostridia; Clostridiales; Lachnospiraceae; Anaerostipes	0.00083	0.000738	0.000622
Firmicutes; Clostridia; Clostridiales; Ruminococcaceae; Oscillospira	0.00083	0.000912	0.000412
Firmicutes; Clostridia; Clostridiales; Ruminococcaceae; Ruminococcaceae UCG-010	0.000579	0.000492	0.000913
Tenericutes; Mollicutes; Mollicutes RF9; uncultured bacterium; uncultured bacterium	0	0.000465	0.001477
Firmicutes; Clostridia; Clostridiales; Lachnospiraceae; Fusicatenibacter	0.000654	0.000702	0.00055
Firmicutes; Clostridia; Clostridiales; Clostridiales vadinBB60 group; gut metagenome	0.000377	0.000492	0.00099
Bacteroidetes; Bacteroidia; Bacteroidales; Porphyromonadaceae; Butyricimonas	0.000654	0.000447	0.00035
Firmicutes; Clostridia; Clostridiales; Lachnospiraceae; Marvinbryantia	0.000503	0.000647	0.00024
Firmicutes; Clostridia; Clostridiales; Lachnospiraceae; Lachnospiraceae UCG-010	0.000302	0.000255	0.000824
Proteobacteria; Gammaproteobacteria; Enterobacteriales; Enterobacteriaceae; Proteus	0	0	0.00136
Proteobacteria; Alphaproteobacteria; Rhodospirillales; Acetobacteraceae; Gluconobacter	0.000604	0.000383	0.000357
Firmicutes; Erysipelotrichia; Erysipelotrichales; Erysipelotrichaceae; uncultured	0.000352	0.000583	0.000333
Bacteroidetes; Bacteroidia; Bacteroidales; Porphyromonadaceae; Coprobacter	0.000528	0.000492	0.000213
Firmicutes; Clostridia; Clostridiales; Ruminococcaceae; Oscillibacter	0.000705	0.000219	0.000295
Tenericutes; Mollicutes; NB1-n; Ambiguous_taxa; Ambiguous_taxa	0.000302	0	0.000836
Firmicutes; Clostridia; Clostridiales; Ruminococcaceae; Flavonifractor	0.000403	0.000419	0.000278
Actinobacteria; Coriobacteriia; Coriobacteriales; Coriobacteriaceae; uncultured	0.000528	0.000264	0.000137
Firmicutes; Erysipelotrichia; Erysipelotrichales; Erysipelotrichaceae; Erysipelotrichaceae UCG-003	0.000377	0.000319	0.000213
Firmicutes; Clostridia; Clostridiales; Ruminococcaceae; Butyricicoccus	7.55E-05	0.000583	0.000227
Proteobacteria; Gammaproteobacteria; Enterobacteriales; Enterobacteriaceae; Escherichia-Shigella	0.000403	0.000264	0.00021
Firmicutes; Clostridia; Clostridiales; Peptostreptococcaceae; Intestinibacter	0.000503	0.000228	0.000131
Proteobacteria; Gammaproteobacteria; Enterobacteriales; Enterobacteriaceae; Citrobacter	0	0.000848	0
Firmicutes; Clostridia; Clostridiales; Lachnospiraceae; Ambiguous_taxa	0	0.000811	0
Firmicutes; Bacilli; Lactobacillales; Enterococcaceae; Enterococcus	0.000478	0.000328	0
Bacteroidetes; Bacteroidia; Bacteroidales; Prevotellaceae; Prevotella 9	0.000277	0.000365	0.000124
Firmicutes; Clostridia; Clostridiales; Ruminococcaceae; Ruminiclostridium 5	0.000252	0.00031	0.000124
Firmicutes; Erysipelotrichia; Erysipelotrichales; Erysipelotrichaceae; Erysipelotrichaceae UCG-004	0.000277	0.000246	0.000089
Firmicutes; Clostridia; Clostridiales; Lachnospiraceae; Lachnospiraceae FCS020 group	0	0.000456	0.000151

Firmicutes; Clostridia; Clostridiales; Lachnospiraceae; Lachnospiraceae NC2004 group	0	0	0.000521
Firmicutes; Clostridia; Clostridiales; Lachnospiraceae; [Eubacterium] oxidoreducens group	0	0	0.000521
Firmicutes; Clostridia; Clostridiales; Clostridiales vadinBB60 group; uncultured organism	0.000252	0.000164	0.000045
Firmicutes; Clostridia; Clostridiales; Ruminococcaceae; Anaerotruncus	0.000176	0.000173	0.00011
Tenericutes; Mollicutes; Mollicutes RF9; __; __	0	0.00021	0.000179
Proteobacteria; Gammaproteobacteria; Pseudomonadales; Moraxellaceae; Enhydrobacter	0.000176	0.000082	0.000079
Firmicutes; Clostridia; Clostridiales; Lachnospiraceae; [Eubacterium] ventriosum group	0	0.000182	0.000076
Lentisphaerae; Lentisphaeria; Victivallales; vadinBE97; uncultured bacterium	0.000050	0.000173	0.000027
Archaea; Euryarchaeota; Methanobacteria; Methanobacteriales; Methanobacteriaceae; Methanobrevibacter	0	0.000182	0.000048
Firmicutes; Clostridia; Clostridiales; Family XIII; Family XIII UCG-001	0	0.000118	0.000096
Firmicutes; Bacilli; Lactobacillales; Streptococcaceae; Streptococcus	0	0.000155	0.000055
Firmicutes; Clostridia; Clostridiales; Lachnospiraceae; Eisenbergiella	0	0.0001	0.000106
Proteobacteria; Deltaproteobacteria; Desulfovibrionales; Desulfovibrionaceae; Bilophila	0	0.0001	0.000096
Firmicutes; Clostridia; Clostridiales; Peptococcaceae; Peptococcus	0	0.000109	0.000079
Proteobacteria; Deltaproteobacteria; Desulfovibrionales; Desulfovibrionaceae; Desulfovibrio	0.000126	0	0.000045
Proteobacteria; Betaproteobacteria; Burkholderiales; Comamonadaceae; Comamonas	0	0.000118	0.000048
Cyanobacteria; Melainabacteria; Gastranaerophilales; Clostridium sp. K4410.MGS-306; Clostridium sp. K4410.MGS-306	0	0.000118	0.000027
Firmicutes; Bacilli; Bacillales; Bacillaceae; Bacillus	0	0.000128	0
Firmicutes; Clostridia; Clostridiales; Lachnospiraceae; Moryella	0.000126	0	0
Firmicutes; Clostridia; Clostridiales; Lachnospiraceae; Tyzzerella	0	0.000055	0.000052
Proteobacteria; Gammaproteobacteria; Pasteurellales; Pasteurellaceae; Haemophilus	0.000101	0	0
Firmicutes; Clostridia; Clostridiales; Family XIII; Family XIII AD3011 group	0	0	0.000072
Firmicutes; Clostridia; Clostridiales; Lachnospiraceae; [Eubacterium] ruminantium group	0	0	0.000072
Bacteroidetes; Bacteroidia; Bacteroidales; Prevotellaceae; Paraprevotella	0	0	0.000065
Firmicutes; Clostridia; Clostridiales; Lachnospiraceae; Lachnospiraceae UCG-008	0	0	0.000052
Proteobacteria; Gammaproteobacteria; Pseudomonadales; Moraxellaceae; Acinetobacter	0	0	0.000052
Unassigned	0.000050	0	0
Bacteroidetes; Bacteroidia; Bacteroidales; Bacteroidales S24-7 group; gut metagenome	0	0	0.000038
Firmicutes; Clostridia; Clostridiales; Ruminococcaceae; Intestinimonas	0	0	0.000038
Firmicutes; Clostridia; Clostridiales; Clostridiales vadinBB60 group; Ambiguous_taxa	0	0	0.000034
Firmicutes; Clostridia; Clostridiales; Ruminococcaceae; Hydrogenoanaerobacterium	0	0	0.000031

Actinobacteria; Coriobacteriia; Coriobacteriales; Coriobacteriaceae; Adlercreutzia	0	0	0.000027
Actinobacteria; Coriobacteriia; Coriobacteriales; Coriobacteriaceae; Slackia	0	0	0.000021
Firmicutes; Clostridia; Clostridiales; Christensenellaceae; uncultured	0	0	0.000021
Firmicutes; Clostridia; Clostridiales; Ruminococcaceae; Caproiciproducens	0	0	0.000021
Bacteroidetes; Bacteroidia; Bacteroidales; Prevotellaceae; Prevotella	0	0.000018	0
Bacteroidetes; Bacteroidia; Bacteroidales; uncultured; Ambiguous_taxa	0	0	0.000017
Firmicutes; Bacilli; Bacillales; Planococcaceae; Kurthia	0	0	0.000017
Firmicutes; Bacilli; Lactobacillales; Leuconostocaceae; Oenococcus	0	0	0.000017
Firmicutes; Clostridia; Clostridiales; Lachnospiraceae; Howardella	0	0	0.000017
Firmicutes; Clostridia; Clostridiales; Clostridiales vadinBB60 group; uncultured Clostridia bacterium	0	0	0.000010
Firmicutes; Erysipelotrichia; Erysipelotrichales; Erysipelotrichaceae; Catenisphaera	0	0	0.000010
Bacteria	0	0	0.000010
Bacteroidetes; Bacteroidia; Bacteroidales; Prevotellaceae; Alloprevotella	0	0	0.000007

Taxa listed in order of high -> low relative abundance.
